# Why Médecins Sans Frontières (MSF) provides safe abortion care and what that involves

**DOI:** 10.1186/s13031-016-0086-5

**Published:** 2016-09-21

**Authors:** Catrin Schulte-Hillen, Nelly Staderini, Jean-François Saint-Sauveur

**Affiliations:** 1 Médecins Sans Frontières International, 78 rue de Lausanne, 1201 Geneva, Switzerland; 2 Médecins Sans Frontières Switzerland, 78 rue de Lausanne, 1201 Geneva, Switzerland; 3 Medicos Sin Fronteras, Carrer Nou de la Rambla 26, 08001 Barcelona, Spain

**Keywords:** Maternal mortality, Abortion, MSF

## Abstract

MSF responds to needs for the termination of pregnancy, including on request (TPR); it is part of the organization’s work aimed at reducing maternal mortality and suffering; and preventing unsafe abortions in the countries where we work. Following the publication of “Why don’t humanitarian organizations provide safe abortion care?” we offer an insight into MSF’s experience over the past few years. The article looks at the legal concerns and proposes that the importance of addressing maternal mortality should replace them and the operational set-up and action organized in a way that mitigates risks. MSF took a policy decision on safe abortion care in 2004; the fact that care did not expand rapidly to relevant MSF projects came as a surprise, reflecting the important weight social norms around abortion have everywhere. The need to engage in an open dialogue with staff, relevant medical actors and at community level became more obvious. Finally the article looks some key lessons that have emerged for the organization as part of the effort to prevent ill health, maternal death and suffering caused by unwanted pregnancy and unsafe abortion.

## Main text

The article “Why don’t humanitarian organizations provide safe abortion services?” [1], published 24 March 2016 in *Conflict and Health*, makes very relevant points about organizations’ disinclination to provide safe abortion care; we commend the authors for their initiative. MSF responds to needs for the termination of pregnancy, including on request (TPR); it is part of the work aimed at reducing maternal mortality and preventing unsafe abortions in the countries where we work [2]. We would like to encourage related reflection by offering insight into MSF’s experience over the past few years.

## Preventing the consequences of unwanted pregnancy and unsafe abortion

Unsafe abortion is one of the world’s main causes of maternal mortality [3] and the only one that is entirely preventable. Unwanted pregnancy and unsafe abortion are important causes of ill health, suffering and death. In 2014, MSF teams treated over 10,000 women and girls presenting with abortion-related complications; they are among the main obstetric complications. From a clinical point of view the distinction between complications related to a miscarriage and those resulting from an unsafe abortion are most often not possible. Based on international evidence [4] and MSF’s own data [5], it may be assumed that anywhere between 50 and 80 % of abortion related complications seen at health facility level are in fact result of an intent to abort and most likely result of an unsafe procedure.

From a health perspective the risks are the same for all women and girls for whom safe abortion care is not available, whether they be a victim of rape, someone who is young and wants to pursue her studies, or a mother who is struggling to provide for her family. In relevant projects MSF strives to provide safe abortion care and by doing so, assumes and accepts potential tensions and repercussions. Data from the past three years reflects that 25 to 35 % of the projects which provided either obstetric care or sexual violence care or both [6] report having ensured provision of safe abortion care in MSF services or having referred patients in need to an alternative quality provider. In absolute numbers, this effort translates into 500 to 1.500 women and girls per year who were granted access to safe care for a requested termination of pregnancy; this represents a small contribution to the real need for safe abortion care in the context were MSF works and where MSF provides skilled birth attendance to about 200.000 women per year.

MSF has made humble advances in the provision of safe abortion care and continues to struggle with internal resistance; however we challenge the authors’ suggestion that “*providing safe abortion to women who become pregnant as a result of rape in war may be a more comfortable place for organizations to begin the discussion of access to safe abortion for all women*”. For medical and humanitarian actors that seems to be a questionable approach. As a medical humanitarian actor, MSF is committed to helping people based on their needs and it is inconceivable that care should be available to some patients and not others in the same context. It contradicts the principle of impartiality, which demands that care be based on need and is provided irrespective of race, religion, gender or political affiliation and point of view [7].

## The importance of addressing maternal mortality needs to replace legal concerns

We agree with the authors of “Why don’t humanitarian organizations provide safe abortion services?” that the blanket statement that abortion is illegal is simply not true. Using the legal context as a general argument to refuse the implementation of safe abortion care is proof of a poor understanding of existing legal provisions. That said, the legal provisions tend to be restrictive and are not easy to navigate. As an example, the establishment of rape is not a medical diagnostic but a legal decision and an exemption based on rape might be hard to defend. Legal procedures are full of hurdles and a woman or girl in need of an abortion may have to justify herself and expose herself to a system that has little consideration for her confidentiality and anguish. A police report may be a requirement in order to access medical care, and a collegial decision of three medical doctors, a prerequisite in some countries to justify termination of pregnancy, may be rendered impossible because no national medical doctors, who are the ones recognized by the authorities for this purpose, are actually available in the context.

While there are encouraging degrees of leniency in most abortion-related legislation, it is not advisable to rely solely on legal “loopholes” as a safety net for action. In countries with very restrictive laws on abortion, legal lenience for certain situations does not mean there is no risk of legal pursuit. The way the law is applied will depend on perception and interpretation. Finally, refusing the provision of safe abortion care based on legal arguments places the importance of “medical necessity” below other considerations.

In the light of the above, it appears that a change of logic is called for. Legal challenges to safe abortion care exist, particularly for termination of pregnancy on request. Are organizations and staff ready to accept them in order to provide patients with medical care that has a direct impact on reducing maternal mortality and suffering?

## Taking responsibility and mitigating risk

MSFs International Council (IC) made a formal decision on safe abortion care in 2004 [8], thus providing full institutional backing for each medical professional providing safe abortion care as part of the organization’s action. The resolution based MSF role in the provision of safe abortion care on the “medical and human needs” of patients; a notion which recognizes the social and political dimension of the issue. In the following years the IC requested assessments [9] of the advances in the provision of safe abortion care and it came as a surprise to many in the MSF leadership that care did not expand to all relevant projects. After an initial increase, the level of availability of safe abortion care in MSF projects stagnated; assessments linked availability mainly to individual commitment, rendering it sensitive to staff turnover. Internal resistance to implementing safe abortion care emerged as the main challenge and with it the need for a stronger policy statement about the medical necessity of safe abortion care, this being the argument where MSF has the strongest legitimacy and a fact that medical staff and all staff working in a medical organization can hardly neglect.

Recently, the policy was revised [10], placing the reduction of maternal mortality and suffering at the core of MSF’s objective and with it, the commitment to preventing the consequences of unwanted pregnancy and unsafe abortion.

By undertaking safe abortion care, MSF has to be prepared for consequences and must work to mitigate risk. First and foremost there is the potential risk to the patient. The woman or girl seeking safe abortion care is at risk of repercussions and needs to be the focus of concern. Ensuring confidentiality is key and must be taken into account in the organization of services.

There is also the risk for the medical staff that are providing the safe abortion care. MSF considers that national staff are particularly exposed to potential repercussions resulting from the provision of safe abortion care in their home country and community, and in places with legal restrictions it is MSF international medical staff who assume the responsibility of providing the necessary care. Contexts with specific security concerns apart, all staff in MSF are expected to guide women and girls who come seeking termination of pregnancy to the medical staff that can best inform them and provide the appropriate care.

When safe abortion care by an alternative quality provider is available, MSF will consider referral as an option and assume the related costs.

MSF’s presence in a country or context, and the capacity to provide medical assistance to people in need, may be challenged as a result of tensions with the authorities, and community perception has to be taken into account. A dialogue with relevant medical actors and at community level needs to be sought in order to share concerns about maternal mortality and abortion-related complications and to reflect MSF’s commitment to working with all concerned to improve women’s and girls health.

Finally it must also not be forgotten that abortion providers may resent MSF for providing free and safe care, and taking business away from them.

## The weight of social norms

MSF still struggles to achieve a change of attitude throughout the organization; change takes time.

There are undeniably strong social norms related to abortion in most societies and a degree of resistance when talking about or dealing with abortion is engrained in most of us, unconsciously or not. The arguments that are put forward by MSF staff at different levels of the organization to explain non-action regarding safe abortion care (such as “there is no need”, “it is too complicated”, “it is not MSF’s role”, “it will put the project into danger”), are often just a reflection of unease, personal reluctance, and a lack of knowledge.

What the last few years have shown us is that in addition to a policy shift, there is the need to actively reach out to staff and to create an environment in which personal feelings, convictions and fears regarding the subject of abortion can be expressed and dialogue is established. In doing so, it is hoped that staff will be able to let personal feelings and professional responsibility co-exist. This is not just about resistance to abortion care though, it is also about pro-choice activism; both are misplaced in an organization that aims to reduce mortality and suffering.

In MSF we see that most medical staff are sensitive to the medical facts and will adapt their attitude once properly informed. Sensitizing medical staff alone however is not sufficient; medical staff needs full support for their efforts from all staff in MSF, particularly from coordinators and project managers. While the commitment to perform safe abortion care can be limited to specific medical professions, the commitment to contributing to MSF’s capacity to respond to abortion-related needs is expected from all.

## Transparency and communication

MSF can undertake the work it does because of support and donations from individuals and other private donors. MSF is committed to transparency. The 2013 MSF International Activity Report included an article entitled “*Addressing Women’s Health Needs*” which talked about the policy on safe abortion care and related medical action [11]. In March 2015, a book and website on women’s health was launched by MSF; it included a chapter on abortion and explained MSF’s policy [12]. Also in 2015, the revised MSF guidelines for essential obstetrics and newborn care [13] were published and included a chapter on the management of “Termination of Pregnancy on Request”. MSF’s position regarding safe abortion care is available publically to anyone who looks for it.

Speaking about abortion is not easy though. When publically asked about safe abortion care, more often than not the person is trying to establish if the organization is doing something “despicable” or “illegal”. They are rarely interested in the fact that MSF is responding to one of the five main causes of maternal mortality and aims to prevent complications and suffering. MSF does not enter into religious, legal, ethical and philosophical debates on the question; as a medical-humanitarian organization, MSF can provide an evidence-based answer for why safe abortion care is a medical necessity.

## Conclusions

For MSF the commitment to safe abortion care is rooted in the concern for maternal mortality, complications and suffering caused by unwanted pregnancy and unsafe abortion - a reality that MSF teams deal with on a daily basis.

Making safe abortion care available to women and girls in need is an ambitious endeavour, and it is not without obstacles and limitations. It engages every part of the organization. MSF has made humble progress over the past few years and some key lessons have emerged: that responsibility towards patients takes priority over other considerations, that social norms regarding abortion must be considered, and that there remains an important knowledge gap, even among MSF staff. An open dialogue with staff, relevant medical actors and at community level is essential to address this and result in a change in attitude. This is likely the most important element to ensuring a safe abortion care for all women and girls in need.

## References

[1] McGinn T, Casey SE (2016). Why don’t humanitarian organizations provide safe abortion services?. Confl Heal.

[2] MSF policy for reproductive health and sexual violence care. 2014. (Internal document)

[3] WHO fact sheet 348. Maternal mortality. 2015. http://www.who.int/mediacentre/factsheets/fs348/en/. Accessed 13 April 2016.

[4] Benson J, Andersen K, Samandari G (2011). Reductions in abortion-related mortality following policy reform: evidence from Romania, South Africa and Bangladesh. Reprod Health.

[5] MSF Reproductive health activity report, 2014 data, page 21-22. Quote: “Data from Leogane-Haiti project allows some insight into the reasons from abortion related complication and their treatment. 77 % of the abortion related complications (incomplete abortion) were successfully treated with manual vacuum aspiration. Among these cases (*n* = 438) 55 % of the women stated that the abortion had been provoked, 41 % women stated a spontaneous abortion and in 4 % of the cases, the abortion was in fact complete” (Internal document).

[6] Projects which provided either obstetric care or sexual violence care or both under direct MSF responsibility: 148 in 2012, 148 in 2013 and 142 in 2014. 2015 data is not yet analysed, but unlikely to vary greatly

[7] MSF Charter and principles. http://www.msf.org/about-msf/msf-charter-and-principles. Accessed 25 Apr 2016.

[8] MSF International Council resolution on abortion, 2004 (Internal document). Quote: “*The availability of safe abortion care should be integrated as part of reproductive health care in all contexts where it is relevant. MSF’s role in termination of pregnancy must be based on the medical and human needs of our patients*”.

[9] L. Bonneville. Update on the Implementation of the IC resolution on reproductive health and abortion, 2007;T. Knudsen. Implementation of abortion care in MSF - Five years after the IC resolution, 2009; C. Schulte-Hillen. Yearly activity report of the SRH working group, 2011. (Internal documents)

[10] Additional statement of the International Board (IB), 2012 Quote: “..*Unsafe abortion and unwanted pregnancy contribute significantly to the burden of ill health, suffering and maternal mortality in the contexts where we work.*” MSF Policy for reproductive health and sexual violence care. 2014 (Internal Document)

[11] MSF International Activity Report. 2013. 16-17. http://activityreport2013.msf.org/#/addressing-womens-health-needs. Accessed 11 Apr 2016.

[12] Because tomorrow needs her, Chapter 4. http://womenshealth.msf.org/. Accessed. 25 Apr 2016.

[13] Essential obstetrics and newborn care, chapter 12. refbooks.msf.org. Accessed 25 Apr 2016.

